# Chromosome engineering of *Escherichia coli* for constitutive production of salvianic acid A

**DOI:** 10.1186/s12934-017-0700-2

**Published:** 2017-05-16

**Authors:** Liang Zhou, Qi Ding, Guo-Zhen Jiang, Zhen-Ning Liu, Hai-Yan Wang, Guang-Rong Zhao

**Affiliations:** 1 Department of Pharmaceutical Engineering, School of Chemical Engineering and Technology, Tianjin University, Tianjin, 300072 China; 2 Key Laboratory of Systems Bioengineering (Ministry of Education), Tianjin University, Tianjin, 300072 China; 3 SynBio Research Platform, Collaborative Innovation Center of Chemical Science and Engineering (Tianjin), Tianjin, 300072 China; 4 College of Chemical and Biological Engineering, Zhejiang University, No. 38 Zhe da Road, Hangzhou, 310027 China; 5 Yangtze River Pharmaceutical Group Co, Ltd., 1 Yangtze River South Road, Taizhou, 225321 China

**Keywords:** Salvianic acid A, *Escherichia coli*, Constitutive promoter, Metabolic engineering, Synthetic biology, Chromosomal engineering

## Abstract

**Background:**

Salvianic acid A (SAA), a valuable natural product from herbal plant *Salvia miltiorrhiza*, exhibits excellent antioxidant activities on food industries and efficacious therapeutic potential on cardiovascular diseases. Recently, production of SAA in engineered *Escherichia coli* was established via the artificial biosynthetic pathway of SAA on the multiple plasmids in our previous work. However, the plasmid-mediated system required to supplement expensive inducers and antibiotics during the fermentation process, restricting scale-up production of SAA. Microbial cell factory would be an attractive approach for constitutive production of SAA by chromosome engineering.

**Results:**

The limited enzymatic reactions in SAA biosynthetic pathway from glucose were grouped into three modules, which were sequentially integrated into chromosome of engineered *E. coli* by λ Red homologous recombination method. With starting strain *E. coli* BAK5, in which the *ptsG*, *pykF*, *pykA*, *pheA* and *tyrR* genes were previously deleted, chassis strain BAK11 was constructed for constitutive production of precursor l-tyrosine by replacing the 17.7-kb *mao*-*paa* cluster with module 1 (P_*lacUV5*_-*aroG* ^*fbr*^-*tyrA* ^*fbr*^-*aroE*) and the *lacI* gene with module 2 (P_*trc*_-*glk*-*tktA*-*ppsA*). The synthetic 5*tacs* promoter demonstrated the optimal strength to drive the expression of *hpaBC*-*d*-*ldh* ^*Y52A*^ in module 3, which then was inserted at the position between *nupG* and *speC* on the chromosome of strain BAK11. The final strain BKD13 produced 5.6 g/L of SAA by fed-batch fermentation in 60 h from glucose without any antibiotics and inducers supplemented.

**Conclusions:**

The plasmid-free and inducer-free strain for SAA production was developed by targeted integration of the constitutive expression of SAA biosynthetic genes into *E. coli* chromosome. Our work provides the industrial potential for constitutive production of SAA by the indel microbial cell factory and also sets an example of further producing other valuable natural and unnatural products.

**Electronic supplementary material:**

The online version of this article (doi:10.1186/s12934-017-0700-2) contains supplementary material, which is available to authorized users.

## Background

Salvianic acid A (SAA, 3-(3′,4′-dihydroxyphenyl)-2-hydroxypropanoic acid), also called danshensu, is the major bioactive ingredient of traditional Chinese herb plant *Salvia miltiorrhiza* (danshen) which is widely used for the prevention and treatment of vascular diseases in clinic [1, 2]. SAA is well-known for its distinguished antioxidant capacity to scavenge the superoxide anion radicals and free hydroxyl radicals, which is even higher than vitamin C [3]. In recent years, SAA has attracted considerable attentions due to its various pharmacological activities, including inhibition of platelet activation and arterial thrombosis [4], alleviation of alcohol-induced acute liver damage [5] and myocardial ischemia injury [6]. Moreover, SAA derivatives, salvianolic acids B and A, and rosmarinic acid, show promising application in medicines and food industries. Salvianolic acid B has already been used to alleviate angina pectoris and treat coronary heart diseases in clinic [7]. Salvianolic acid A has been approved by China Food and Drug Administration (CFDA) into phase I clinical trial. Rosmarinic acid could be used for food preservation as a natural antioxidant to substitute synthetic antioxidant like butylated hydroxytoluene [8]. Notably, conjugates of SAA with cysteine show better vascular-protective effect than SAA [9].

**Fig. 1:**
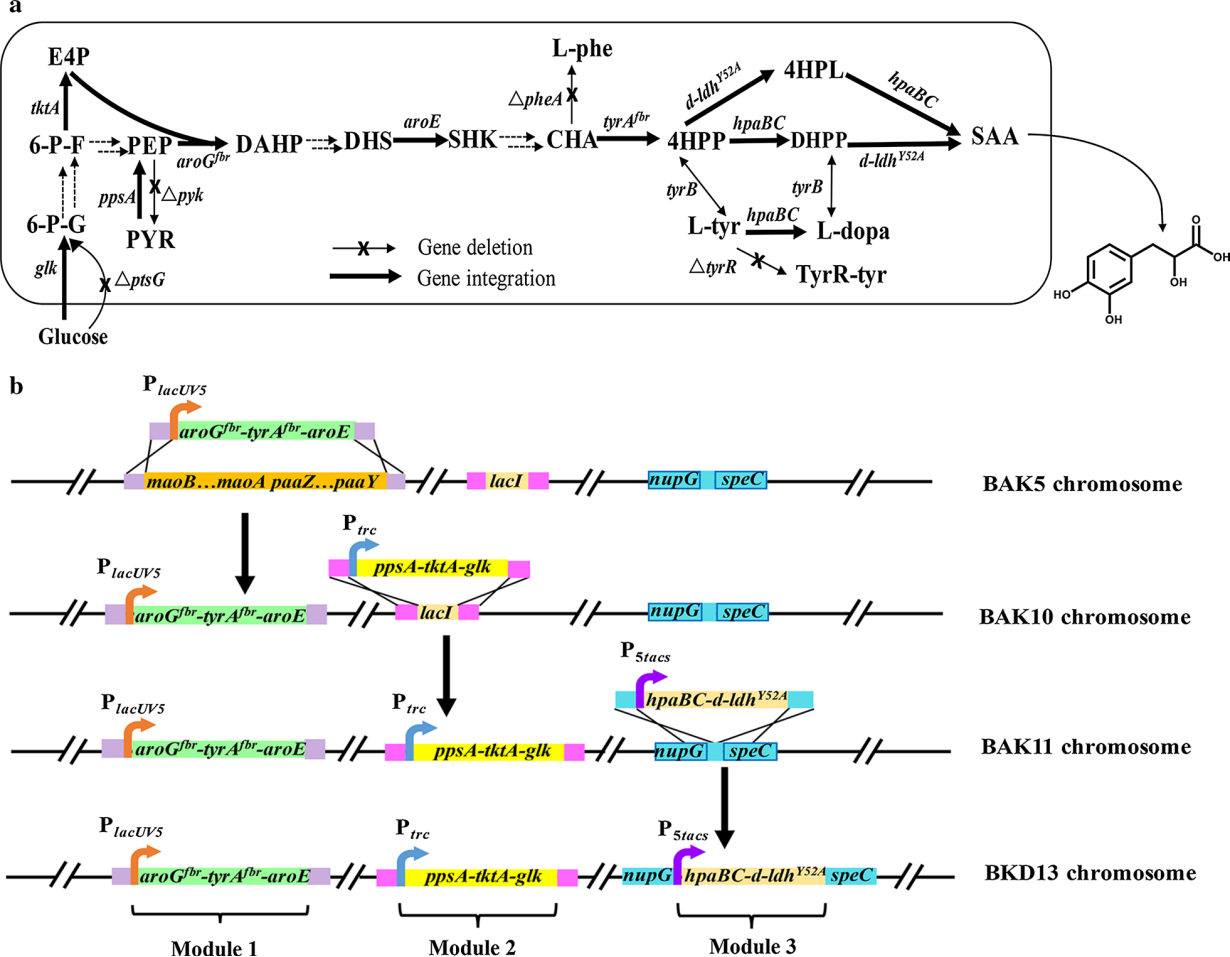
Chromosomal engineering for constitutive production of salvianic acid A (SAA) in *E. coli*. **a** The artificial synthetic pathway of SAA from glucose. **b** The targeted integration of three modules into chromosome of engineered strains. In-frame deletion of the *ptsG*, *pykF*, *pykA*, *pheA* and *tyrR* genes were not shown in chromosome of strain BAK5. *6‐P‐G* 6‐phosphate‐glucose, *6‐P‐F* 6‐phosphate‐fructose, *E4P* erythrose‐4‐phosphate, *PEP* phosphoenolpyruvate, *PYR* pyruvic acid, *DAHP* 3‐deoxy‐d‐arabino‐heptulosonate‐7‐phosphate, *DHS* 3‐dehydroshikimic acid, *SHK* shikimic acid, *CHA* chorismic acid, *l* *-Phe* l-phenylalanine, *4‐HPP* 4‐hydroxyphenylpyruvic acid, *l* *-Tyr* l-tyrosine, *DHPP* 3,4-Dihydroxyphenylpyruvate, *4-HPL* 4-Hydroxyphenyllactate, *TyrR-tyr* TyrR-tyrosine DNA-binding transcriptional repressor, *SAA* salvianic acid A

Although SAA could be extracted from root of *S. miltiorrhiza*, tiny amount of SAA in roots (0.045%) restricts its application [10]. The chemical synthesis of SAA suffers from intractable enantioselectivities for large-scale production [11]. An alternative route for SAA production was developed previously in our laboratory via metabolic engineering of *Escherichia coli* [12, 13] (Fig. 1a). In order to intensify the availability of PEP, block the competitive biosynthesis of l-phenylalanine and eliminate the transcriptional repression of genes in l-tyrosine biosynthetic pathway, the *ptsG*, *pykF*, *pykA*, *pheA* and *tyrR* genes were deleted, and l-tyrosine overproducing strain BAK5 was obtained. For efficient production of SAA, strain BAK5 harbored three extra expression plasmids: middle-copy-number plasmid (30–40 copies) carrying module 1 (*aroG* ^fb*r*^-*tyrA* ^*fbr*^-*aroE*) and module 2 (*ppsA*-*tktA*-*glk*), middle-copy-number plasmid (~20 copies) carrying module 3 (*hpaBC*-*d*-*ldh* ^*Y52A*^), and low-copy-number plasmid carrying T7 RNA polymerase gene [12]. Despite this available alternative, SAA production by plasmid-mediated strain had serious drawbacks. Expensive isopropyl-β-d-thiogalactopyranoside (IPTG) was necessary to induce the expression of target genes. Additional antibiotics to maintain the genetic stability were harmful to cell growth and environmentally unfriendly [14]. Taken together, overexpressing target genes on multiple plasmids has become a barrier for industrial-scale production of natural product SAA.

Synthetic biology and metabolic engineering are promising strategies to deal with those obstacles by reconstructing inducer-free and plasmid-free strains. The constitutive promoters, the core elements for metabolic engineering, have been paid more attention to allowing inducer-free and continuous gene expression as well as low production cost [15]. Native constitutive promoters have been widely employed for producing bio-based chemicals [16–18] and natural products [19–22] by tuning target gene expression in engineered yeasts. In *E. coli*, the conventional *T7* promoter has always been used for heterologous protein expression by additional inducer IPTG, but the constitutive promoters showed better performance for production of natural and non-natural products, which are adapted to the growth of host cells. The native constitutive *gap* promoter was more suitable for the heterologous expression of the *4* *cl* and *sts* genes in *E. coli* than the inducible *T7* promoter, resulting in high production of resveratrol [23]. The P_*L*_ derivative promoters could constitutively drive target gene expression in *E. coli* to maximize the desired phenotypes and benefit for effective production of lycopene [24], 2,3-butanediol [25] and human growth hormone receptor antagonist [26]. In addition, chromosomal integration of the target gene would eliminate the use of plasmids in host cells and relieve the metabolic burden. Excellent examples were high-yielding production of artemisinic acid, a precursor of artemisinin, and lycopene in baking yeast [27, 28]. The plasmid-free *E. coli* strains could stablely produce astaxanthin [29], shikimic acid [30] and β-carotene [31].

In this study, *E. coli* BAK5 [12] was used as the start strain. Limited enzymatic reactions of SAA synthetic pathway from glucose in engineered *E. coli* were grouped into three modules as in previous work [12]. Modules 1 and 2 were integrated into the chromosome of strain BAK5, and strain BAK11 for overproducing l-tyrosine without any plasmids was constructed (Fig. 1b). The optimal promoter for driving module 3 was screened out by fine tuning constitutive expression, and integrated into chromosome of strain BAK11, a plasmid-free and inducer-free *E. coli* strain BKD13 for constitutive production of SAA was constructed (Fig. 1b). Finally, fed-batch fermentation was taken out and engineered strain BKD13 produced 5.6 g/L of SAA in 60 h. To our knowledge, this is the first report describing SAA production by engineered *E. coli* without addition of any inducers and antibiotics.

## Methods

### Bacterial strains, plasmids and reagents

The bacterial plasmids and strains used in this study were listed in Tables 1 and 2, respectively. All primers used in this study were summarized in Additional file 1: Table S1). *E. coli* DH5α was employed for all gene cloning work and *E. coli* BW25113 derivatives were used to construct SAA producing strains. The primers and long DNA fragments were synthesized by GENEWIZ (Suzhou, China). Methanol and acetate (HPLC grade) were purchased from Concord Tech (China). All other chemicals and reagents used in the experiment were of analytical grade commercially available. SAA (98% purity) was purchased from Xi’an Honson Biotechnology Company (China). l-tyrosine (99.9% purity) and l-dopa (99.9% purity) were purchased from Dingguo Biotech (China). DNA Polymerase of Phanta Super Fidelity and Taq for PCR were purchased from Vazyme (Nanjing, China). T4 DNA ligase and restriction endonucleases were purchased from Thermo Scientific (Beijing, China). The plasmids were constructed by ligating the PCR products and plasmids, which were all digested by the same restriction endonucleases. Successful recombinant plasmids were confirmed by colony PCR and DNA sequencing.

**Table 1:** Plasmids used in this study

Plasmids	Characteristics	Source
pKD3	FRT (FLP recognition target) sites; Cm^R^	[32]
pKD46	Red recombinase expression vector; Amp^R^	[32]
pCP20	FLP expression vector; Amp^R^, Cm^R^	[32]
pACYCDuet-1	p15A ori; Cm^R^	Novagen
pYBT5	pBldgbrick 1 with P_*lacUV5*_-*aroG* ^*fbr*^-*tyrA* ^*fbr*^-*aroE,* P_*trc*_-*ppsA*-*tktA*-*glk*	[12]
pYBD4	pCDFDuet-1 with *hpaBC* and *d*-*ldh* ^*Y52A*^	[12]
pZL	p15A ori; Cm^R^	This study
pZL1	pZL with fragment 1	This study
pZL2	pZL with fragment 2	This study
pZL3	pZL with P_*BBa*-*J23100*_-*hpaBC* and P_*BBa*-*J23100*_-*d*-*ldh* ^*Y52A*^	This study
pZL4	pZL with P_*tac*_-*hpaBC* and P_*tac*_-*d*-*ldh* ^*Y52A*^	This study
pZL5	pZL with P_*tac*_-*hpaBC* and P_*BBa*-*J23100*_-*d*-*ldh* ^*Y52A*^	This study
pZL6	pZL with P_*BBa*-*J23100*_-*hpaBC and* P_*tac*_-*d*-*ldh* ^*Y52A*^	This study
pZL7	pZL with fragment 3	This study
pZL8	pZL7 with *rrnB* P1 promoter	This study
pZL9	pZL with P_*5tacs*_-*hpaBC*- *d*-*ldh* ^*Y52A*^	This study
pZL10	pZL with P_*rrnB P1*_-*hpaBC*- *d*-*ldh* ^*Y52A*^	This study

**Table 2:** Strains used in this study

Strains	Characteristics	Source
BAK5	BW25113 Δ*ptsG*, Δ*tyrR*, Δ*pykA*, Δ*pykF*, Δ*pheA*	[12]
BAK10-1	BAK5 Δ*mao*-*paa* cluster::P_*lacUV5*_-*aroG* ^*fbr*^-*tyrA* ^*fb*r^-*aroE*-*Chl*	This study
BAK10	BAK5 Δ*mao*-*paa* cluster::P_*lacUV5*_-*aroG* ^*fbr*^-*tyrA* ^*fb*r^-*aroE*	This study
BAK11-1	BAK10 Δ*lacI*::P_*trc*_-*ppsA*-*tktA*-*glk*-*Chl*	This study
BAK11	BAK10 Δ*lacI*::P_*trc*_-*ppsA*-*tktA*-*glk*	This study
BKD5	BAK5 with pYBH1, pYBT5 and pYBD4	[12]
BKD7	BAK11 with pZL3	This study
BKD8	BAK11 with pZL5	This study
BKD9	BAK11 with pZL6	This study
BKD10	BAK11 with pZL4	This study
BKD11	BAK11 with pZL10	This study
BKD12	BAK11 with pZL9	This study
BKD13-1	BAK11 *nupG*::P_5*tacs*_-*hpaBC*-*d*-*ldh* ^*Y52A*^-*Chl*	This study
BKD13	BAK11 *nupG*::P_5*tacs*_-*hpaBC*-*d*-*ldh* ^*Y52A*^	This study

### Construction of SAA-producing expression vectors with constitutive promoters

Four constitutive promoters, P_*BBa*-*J23100*_, P_*tac*_, P_5*tacs*_ and P_*rrnB P1*_, were used to construct expression vectors. Three fragments (F1, F2 and F3) containing multiple cloning sites*, BBa*-*B0015* terminator were designed and synthesized to simplify the vector construction (Additional file 1: Table S2). In order to achieve the maximum translation level, the synthetic 5′-untranslated region (5′-UTR) sequences of the *hpaBC* and *d*-*ldh* ^*Y52A*^ genes were predicted by the UTR Designer (http://sbi.postech.ac.kr/utr_designer) and designed in primers hpaBC F and d-ldh^Y52A^ F directly. Plasmid pACYCDuet-1 was digested with *Apa*I and *Xho*I, and the fragment containing the p15A ori and Cm^R^ was served as the skeleton vector pZL.

To construct plasmids pZL3, 4, 5 and 6 for bicistron expression of the *hpaBC* and *d*-*ldh* ^*Y52A*^ genes, pZL ligated with fragment F1 containing two *BBa*-*J23100* promoters and fragment F2 containing two *tac* promoters at *Apa*I*/Xho*I, respectively, generating pZL1 and pZL2. Then pZL1 and pZL2 ligated with the *hpaBC* gene at *Hin*dIII/*Spe*I and the *d*-*ldh* ^*Y52A*^ gene at *Eco*RI/*Bam*HI, which were amplified from plasmid pYBD4 with primers hpaBC F/R and d-ldh^Y52A^ F/R, respectively, generating pZL3 and pZL4. The P_*BBa*-*J23100*_-*hpaBC* of pZL3 was replaced with P_*tac*_-*hpaBC* of pZL4 at *Apa*I/*Spe*I to generate pZL5. The P_*BBa*-*J23100*_-*d*-*ldh* ^*Y52A*^ of pZL3 was replaced with P_*tac*_-*d*-*ldh* ^*Y52A*^ of pZL4 at *Spe*I/*Bam*HI to generate pZL6.

To construct plasmids pZL9 and pZL10 for expressing the *hpaBC* and *d*-*ldh* ^*Y52A*^ genes as one operon, pZL ligated with F3 containing the 5*tacs* promoter digested with *Apa*I*/Xho*I, generating pZL7. The *rrnB* P1 promoter cloned from BAK5 genome with primers rrnB F/R was inserted into pZL7 digested with *Apa*I/*Hin*dIII, generating plasmid pZL8. Then pZL7 and pZL8 ligated with the *hpaBC* and *d*-*ldh* ^*Y52A*^ genes, generating pZL9 and 10, respectively.

### Modular integration into chromosome

The integration strains were derived from strain BAK5 by using the λ Red homologous recombination method [32]. Module 1 replaced the *mao*-*paa* cluster, and module 2 replaced the *lacI* gene. Module 3 was integrated at the locus between *nupG* and *speC*. The three recombinated fragments for targeted integration of corresponding modules (ydbL-P_lacUV5_-aroG^fbr^-tyrA^fbr^-aroE-Chl-ydbA for module 1, lacZ-P_trc_-ppsA-tktA-glk-Chl-mhpR for module 2, nupG-P_5tacs_-hpaBC-d-ldh^Y52A^-Chl-speC for module 3) were constructed by overlapping extension PCR. Here, construction process for module 1 integrating fragment as example was demonstrated in Additional file 1: Figure S1. Module 1 (P_lacUV5_-aroG^fbr^-tyrA^fbr^-aroE) was amplified from pYBT5 employing primers M1 F/R. The ydbL fragment (500 bp upstream) and the ydbA fragment (500 bp downstream) as homologous arms were cloned from BAK5 genome with primers ydbL F/R and ydbA F/R, respectively. Plasmid pKD3 was used as a template to clone the chloramphenicol resistance cassette employing primers Chl F/R. The ydbL fragment and module 1 were assembled into the ydbL-M1 fragment by overlapping extension PCR, and the chloramphenicol resistance cassette and the *speC* fragment were assembled into the Chl-ydbA fragment. The final fragment ydbL-M1-Chl-ydbA was generated by combining fragments ydbL-M1 with Chl-ydbA, and then electrotransformed into strain BAK5 which contained plasmid pKD46. The positive clone was confirmed by PCR. Afterwards, the chloramphenicol resistance was eliminated with the help of plasmid pCP20, which was further verified by PCR, and the final strain BAK10 was obtained. The same procedure was performed for the integration of module 2 and module 3, respectively.

### Fermentation media and cultivation conditions

*Escherichia coli* cells were cultivated in Luria broth (LB) for strain maintenance and seed preparation. Modified MOPS (morpholinepropanesulfonic acid) medium containing 1× MOPS minimal salt [33], yeast extract (1 g/L) and glucose (5 g/L) was used for the production of l-tyrosine. The cultivation was conducted at 37 °C and 220 rpm with 50 mL of modified MOPS medium in 250 mL of shake flasks for 24 h. YM9 medium contained glucose (5 g/L), Na_2_HPO_4_ (6 g/L), KH_2_PO_4_ (3 g/L), NH_4_Cl (1 g/L), NaCl (0.5 g/L), CaCl_2_ (17 mg/L), MgSO_4_ (58 mg/L) and yeast extract (1 g/L) was used for the production of SAA. The cultivation was conducted at 30 °C and 220 rpm with 50 mL of YM9 medium in 250 mL of shake flasks for 24 h. Appropriate antibiotics were added in medium when necessary: ampicillin (50 μg/mL) and chloramphenicol (20 μg/mL).

For bioreactor fermentation, seed culture (~400 mL) was inoculated into a 5 L fermenter (Bailun, Shanghai) containing 2.1 L fermentation medium, generating an initial OD_600_ of ~0.6. The fermentation medium contained glucose (7.5 g/L), Na_2_HPO_4_ (6.8 g/L), KH_2_PO_4_ (8.5 g/L), NH_4_Cl (3 g/L), NaCl (0.5 g/L), CaCl_2_·2H_2_O (0.07 g/L), MgSO_4_·7H_2_O (1 g/L), yeast extract (5 g/L). The pH was maintained at 7.0 by automatic addition of 10 M NaOH solution. The fermentation was performed at 30 °C with a 2.5 L/min airflow, and the dissolved oxygen (DO) level was controlled at 30% (v/v) by changing the agitation speed from 300 to 600 rpm automatically. The feeding solution contained 500 g/L glucose and 60 g/L yeast extract. Samples of fermentation broth were periodically withdrawn for analysis. The fermentation experiments were carried out in triplicates.

### Analytical methods

Cell growth was determined by measuring the optical density (OD_600_) using a TU-1810 spectrophotometer. The concentration of residual glucose was quantified by a biosensor SBA-90 (Biology Institute of Shandong Academy of Sciences, China). To measure l-tyrosine, the sample was prepared as previously described [12]. To measure SAA and l-dopa, the broth sample was centrifuged directly and the supernatant was filtered through 0.22 μm syringe filter. All the metabolites were analyzed using Agilent 1200 HPLC system (LabAlliance Corp, USA) equipped with a C18 column (250 mm × 4.6 mm, 5 μm, Bonna-Agela, China) and a DAD detector (Agilent). The column temperature was set at 25 °C. 10 μL of sample was injected to the HPLC system for analysis. SAA, l-tyrosine and l-dopa were quantified at 281 nm. The mobile phase was methanol–water–acetate (20:80:0.1, v/v/v) and the flow rate was set at 1 mL/min. Identification and quantitation of compounds were verified by comparison of retention time and using a standard curve, which the R^2^ coefficient was higher than 0.99.

## Results and discussion

### Construction of plasmid-free chassis strain for constitutive production of precursor l-tyrosine

l-tyrosine is precursor for SAA biosynthesis (Fig. 1a). Accordingly, sufficient supplement of l-tyrosine facilitates the production of SAA. The biosynthetic pathway of l-tyrosine is tightly regulated by l-tyrosine feedback and transcription repression [34]. A variety of metabolic engineering approaches have been use to improve l-tyrosine production by deleting repressing gene *tyrR* and overexpressing feedback-resistant genes *aroG* ^*fbr*^ and *tyrA* ^*fbr*^, and other genes of limited steps on plasmids [35, 36]. Plasmid-mediated l-tyrosine producer strains needed the addition of corresponding antibiotics and IPTG to control gene overexpression of interest [37–39]. Although feedback-resistant genes *aroG* ^*fbr*^ and *tyrA* ^*fbr*^ were integrated into the *tyrR* locus of *E. coli* chromosome, inducer IPTG was required for l-tyrosine fermentation as the *T7* promotor was used [40].

**Fig. 2:**
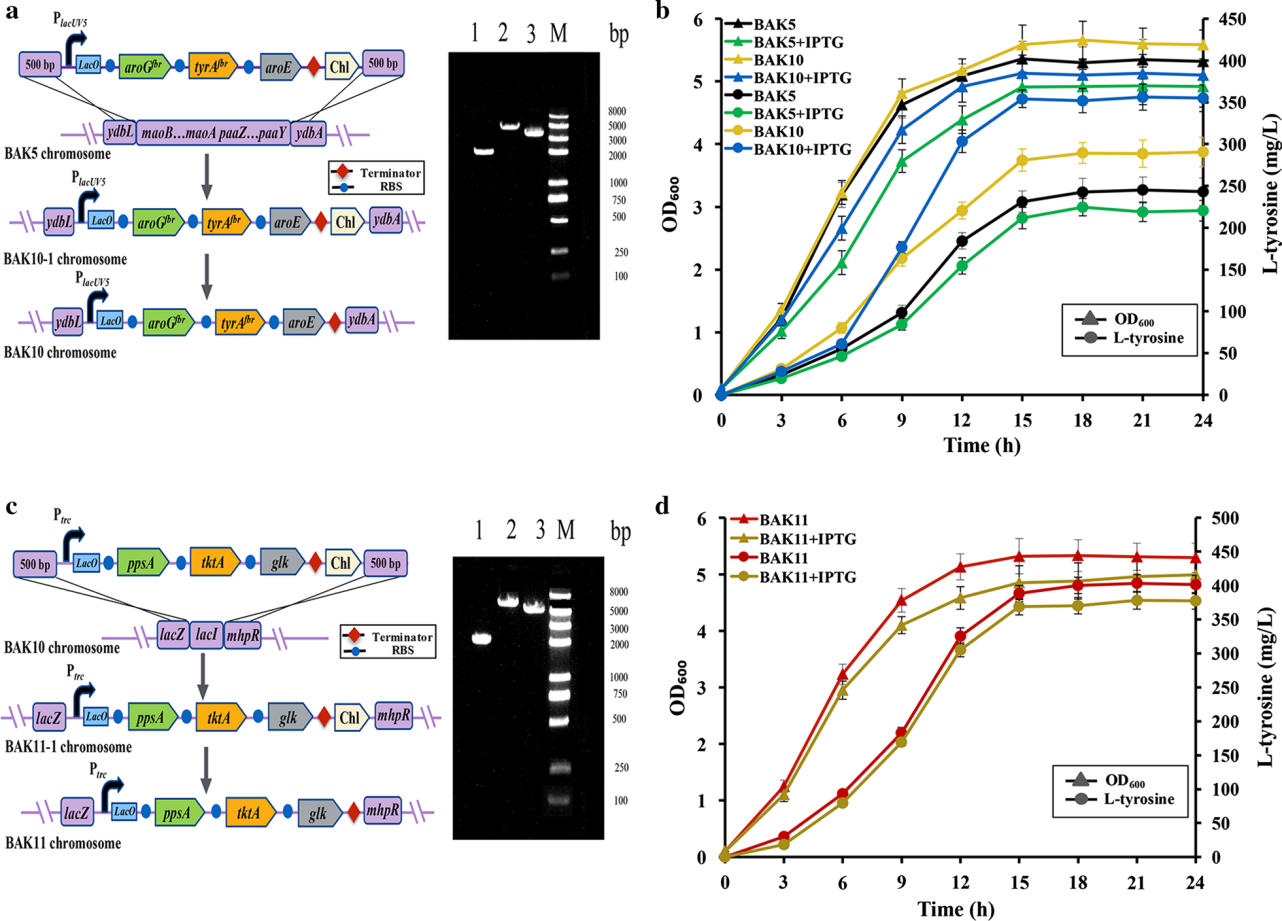
Construction of plasmid-free chassis strain BAK11 to produce l-tyrosine constitutively. **a** Replacement of the *mao*-*paa* cluster with module 1. *Lane 1* The *paaZ* gene of the *mao*-*paa* cluster in strain BAK5 with primers paaZ F/R; *lane 2* ydbL-M1-Chl-ydbA fragment in strain BAK10-1 with primers ydbL F and ydbA R; *lane 3* ydbL-M1-ydbA fragment in strain BAK10 with primers ydbL F and ydbA R; M: DNA marker. **b** Cell growth and l-tyrosine production of strains BAK5 and BAK10. 0.1 mM IPTG was added into the medium when needed. **c** Replacement of the *lacI* gene with module 2. *Lane 1* ydbL-lacI-ydbA in strain BAK10; *lane 2* ydbL-M2-Chl-ydbA fragment in strain BAK11-1; *lane 3* ydbL-M2-ydbA fragment in strain BAK11; primers lacZ F and mhpR R were used for all PCR verification. M: DNA marker. **d** Cell growth and l-tyrosine production of strain BAK11. 0.1 mM IPTG was added into the medium when needed

In previous work, we constructed an engineered l-tyrosine overproducing *E. coli* strain BAK5 with a plasmid overexpressing module 1 and module 2. Module 1 containing *aroG* ^*fbr*^-*tyrA* ^*fbr*^-*aroE* and module 2 consisting of *ppsA*-*tktA*-*glk* were driven by the *lacUV5* and *trc* promoters (Fig. 1b), respectively. In order to eliminate the drawbacks of plasmid-mediated system, module 1 and module 2 were integrated into the chromosome of strain BAK5 for constitutive production of precursor l-tyrosine. The *mao*-*paa* cluster involved in the degradation of aromatic acids [41] was chosen as the target site to integrate module 1. The fragment ydbL-P_lacUV5_-aroG^fbr^-tyrA^fbr^-aroE-Chl-ydbA was assembled by overlapping extension PCR, and replaced the long *mao*-*paa* cluster (~17.7 kb) on chromosome of strain BAK5 by λ Red homologous recombination method [32] as described in section of “Methods”. Successful replacement of the *mao*-*paa* cluster with module 1 was verified by the colony PCR, and strain BAK10 was generated (Fig. 2a).

In order to test integrative effect of module 1 on the production of l-tyrosine, the shake fermentation was carried out. As shown in Fig. 2b, l-tyrosine titer of strain BAK10 was 320.6 mg/L after 24 h of cultivation without IPTG induced, increasing 31.8% than that of strain BAK5. When inducer IPTG (0.1 M) was added into fermentation medium, the production of l-tyrosine had a 22.1% increment, indicating that the expression of module 1 was partially repressed by the negative regulatory gene *lacI* due to the existence of *lacO* in *lacUV5* promoter region of module 1, we speculated that deleting *lacI* gene would further improve the production of l-tyrosine as well as eliminate the supplementation of inducer IPTG. Thus, we replaced *lacI* gene with module 2, and generated strain BAK11 (Fig. 2c). The l-tyrosine titer of BAK11 was 401.5 mg/L without the induction of IPTG (Fig. 2d), increasing 65.1 and 25.2% than those of strains BAK5 and BAK10, respectively. We also investigated whether IPTG could still regulate l-tyrosine production in strain BAK11. After induction with additional IPTG, l-tyrosine production of strain BAK11 was slightly decreased, along with the inhibition of cell growth (Fig. 2d), which might be resulted from the cellular toxicity of inducer IPTG [42]. Deletion of *lacI* gene completely eliminated the repression of LacI, and strain BAK11 constitutively produced precursor l-tyrosine without IPTG induction.

### Screening promoter for constitutive expression of *hpaBC* and *d*-*ldh*^*Y52A*^ to produce SAA

In the downstream of artificial biosynthetic pathway of SAA, module 3 contains the *hpaBC* and *d*-*ldh* ^*Y52A*^ genes, which code 4-hydroxyphenylacetate 3-hydroxylase and d-lactate dehydrogenase, respectively, and catalyze the reactions of 4HPP to SAA via two putative routes (Fig. 1a). To construct inducer-free SAA producing strain, promoters for the constitutive expression of the *hpaBC* and *d*-*ldh* ^*Y52A*^ genes were considered. The constitutive promoter *BBa*-*J23100* has the strongest strength among BBa series of promoters in the Anderson promoter library (Registry of Standard Biological Parts, http://parts.igem.org), and has been used to metabolically engineer *E. coli* for the constitutive production of l-tyrosine [43], butanol [44] and cadaverine [45]. In addition, hybrid *tac* promoter, derived from the *trp* and *lacUV5* promoters, is well-known for the 2–7 folds higher strength than the parent promoters [46]. The *tac* promoter has been popularly employed in metabolic engineering [47, 48]. Thus, two strong constitutive promoters *BBa*-*J23100* and *tac* were chosen for expression of the *hpaBC* and *d*-*ldh* ^*Y52A*^ genes as bicistron. Moreover, according to the N-terminal coding sequences of the *hpaBC* and *d*-*ldh* ^*Y52A*^ genes, 25-bp sequences of 5′-UTR were designed to achieve the the maximum translation level using UTR Designer [49]. The *BBa*-*J23100* and *tac* promoters followed by the 5′-UTR were put in the front of the *hpaBC* and *d*-*ldh* ^*Y52A*^ genes, and four expression plasmids were generated (Additional file 1: Figure S2), after being introduced into strain BAK11, respectively, strains BKD7, BKD8, BKD9 and BKD10 were constructed.

**Fig. 3:**
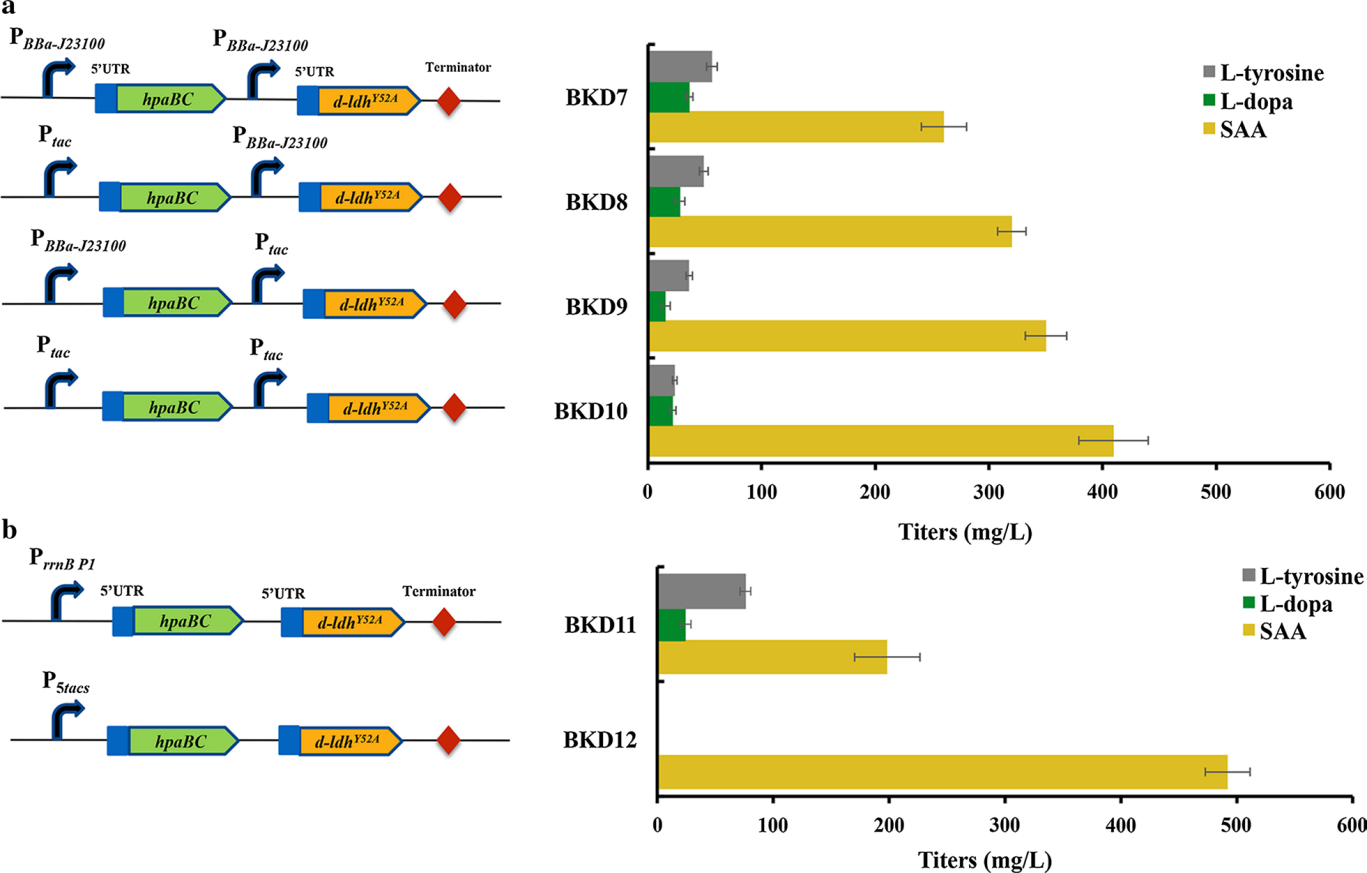
Screening optimal promoter for constitutive expression of *hpaBC* and *d*-*ldh* ^*Y52A*^ for production of SAA. **a** The *hpaBC* and *d*-*ldh* ^*Y52A*^ were expressed as bicistron under the control of the *tac* and *BBa*-*J23100* promoters. **b** *hpaBC*-*d*-*ldh* ^*Y52A*^ was expressed as one operon under the control of the 5*tacs* and *rrnB P1* promoters, respectively

To test expression efficiency of the *hpaBC* and *d*-*ldh* ^*Y52A*^ genes under the control of the *BBa*-*J23100* and *tac* promoters, the production of SAA in fermentation broth was analyzed by HPLC (Additional file 1: Figure S3). As shown in Fig. 3a, among four expression patterns, the *BBa*-*J23100* promoter in strain BKD7 gave the lowest titer of SAA (260.4 mg/L), while the *tac* promoter in strain BKD10 made the highest titer of SAA (409.5 mg/L), the combinations of the *BBa*-*J23100* and *tac* promoters led to the moderate production of SAA. Notably, the broth turned brown or dark after 30 h fermentation. We suspected that the intermediate l-dopa could be accumulated from l-tyrosine and further converted into melanin by HpaBC [50]. As shown in Fig. 3a, l-dopa and l-tyrosine were detected in broth of four strains BKD7, BKD8, BKD9 and BKD10, and the higher accumulation of l-dopa and l-tyrosine, the lower production of SAA, indicating that the expression of the *hpaBC* and *d*-*ldh* ^*Y52A*^ genes driven by either the *tac* or *BBa*-*J23100* promoter was inefficient for SAA production.

The *rrnB P1* promoter was considered as a super constitutive promoter composing of a core promoter, a cis-acting DNA sequence and a trans-acting transcription factor-binding site, and has a major role in high-level synthesis of rRNA during exponential growth of *E. coli* cells [51]. Although the *tac* promoter is stronger than the *BBa*-*J23100* promoter, one copy *tac* promoter might not be enough to express target genes for SAA production (Fig. 3a). The tandem repetitive promoter was more powerful than unrepetitive one for gene expression [52]. Five repetitive *tac* core promoter had suitable strength for transcription control and allowed high production of polyhydroxybutyrate in *E. coli* [53]. Here, the synthetic 5 × *tac* (designated as 5*tacs*) and *rnnB P1* promoters were employed to drive expression of the *hpaBC*-*d*-*ldh* ^*Y52A*^ as monocistron operon (Additional file 1: Figure S2). As shown in Fig. 3b, strain BKD12 (with P_5*tacs*_) produced 492.2 mg/L of SAA, 1.2-fold and 1.5-fold higher than strains BKD10 and BKD11 (with P_*rrnB P1*_, 198.5 mg/L), respectively. We noticed that broth of strain BKD11 turned brown at 27 h fermentation, while broth of strain BKD12 did not. Interestingly, intermediates l-tyrosine (76.1 mg/L) and l-dopa (24.5 mg/L) were detected in broth of strain BKD11, but not in broth of strain BKD12, consistent without the observation of brown color. It indicated that the *hpaBC*-*d*-*ldh* ^*Y52A*^ under the control of the 5*tacs* promoter could completely convert l-tyrosine to SAA.

### Chromosomal integration of module 3 for plasmid-free production of SAA

**Fig. 4:**
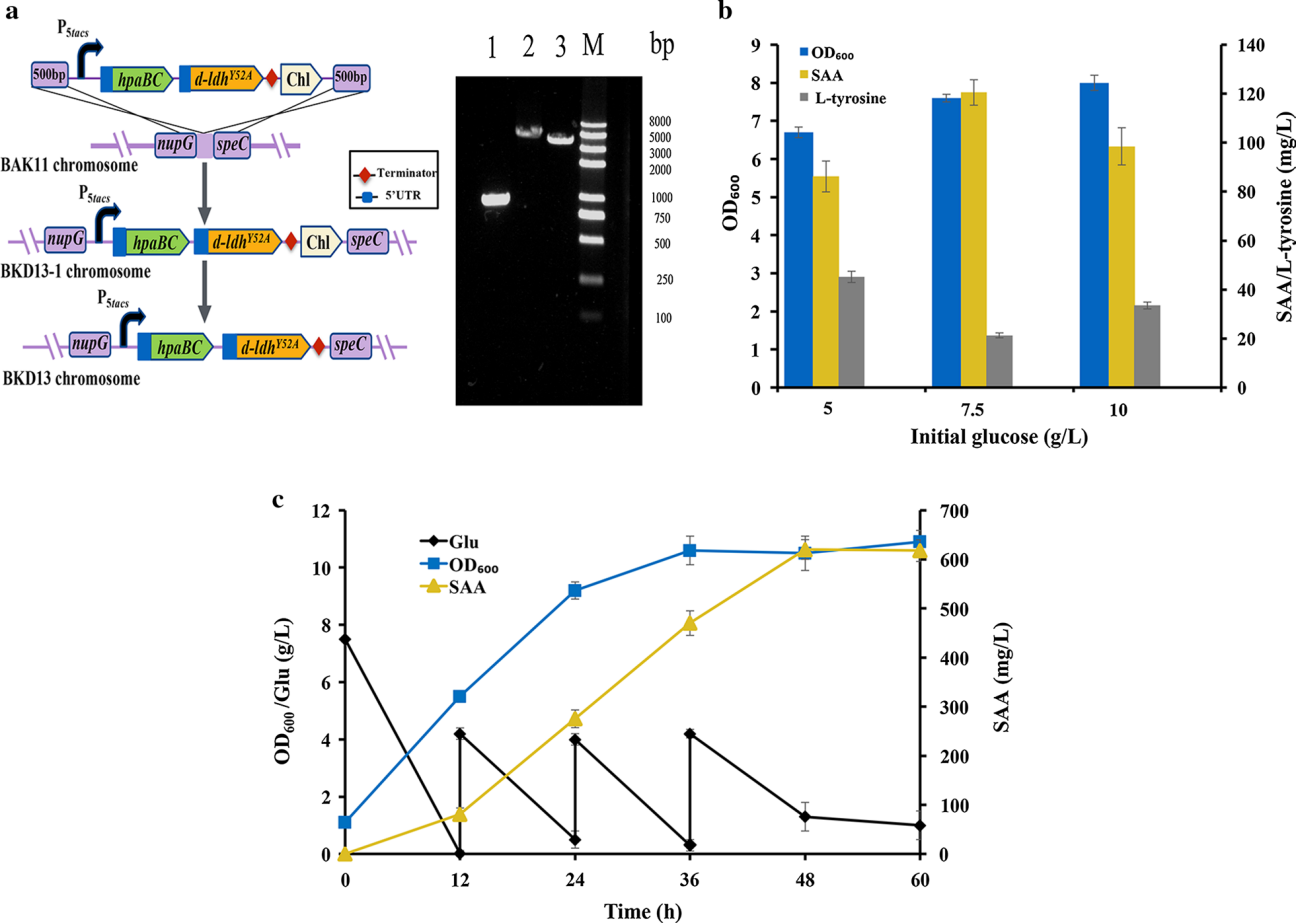
Construction of plasmid-free and inducer-free strain BKD13 for constitutive production of SAA. **a** Integration of module 3 into the locus between *nupG* and *speC*. *Lane 1* nupG-speC fragment in strain BAK10; *lane 2* nupG-M3-Chl-speC fragment in strain BAK11-1; *lane 3* nupG-M3-speC fragment in strain BAK11; primers nupG F and speC R were used for all PCR verification. M: DNA marker. **b** The OD_600_ and titer of BKD13 with different initial glucose concentration. **c** Fed‐batch fermentation of BKD13 with glucose in shake flasks

In order to construct plasmid-free strain, chromosomal integration of module 3 was further adopted. The expression cassette of P_*5tacs*_-*hpaBC*- *d*-*ldh* ^*Y52A*^ in module 3 was integrated at the position between *nupG* and *speC*, which was demonstrated to be the most transcriptionally active for the expression of inserted gene [54], and then plasmid-free strain BKD13 was constructed (Fig. 4a).

To estimate the constitutive production behavior of engineered strain BKD13, preliminary fermentation was conducted in shake flasks with different concentrations of the initial glucose. As shown in Fig. 4b, 86.2 mg/L of SAA was obtained with 5 g/L of glucose, and 45.2 mg/L of l-tyrosine was accumulated in broth (Fig. 4b). With 7.5 g/L of the initial glucose, the SAA titer was increased to 120.5 mg/L and l-tyrosine titer was decreased to 21.3 mg/L, while SAA was decreased and l-tyrosine was increased with higher initial glucose (10 g/L) (Fig. 4b). However, no l-dopa was detected in broth.

In order to testify the potential of strain BKD13 for SAA production, the fed-batch fermentation of SAA was also carried out in shake flasks. As shown in Fig. 4c, with feeding glucose, the cell growth of strain BKD13 entered the stationary phase at 36 h and the SAA production was gradually accumulated to 620.15 mg/L at 48 h. Additionally, there were minor amounts of l-tyrosine (6.5 mg/L) and l-dopa (2.9 mg/L) in fermentation broth. The results indicated that the chromosomal integration of module 3 was efficient to produce SAA.

### Fed‐batch fermentation for constitutive production of SAA in 5 L bioreactor

**Fig. 5:**
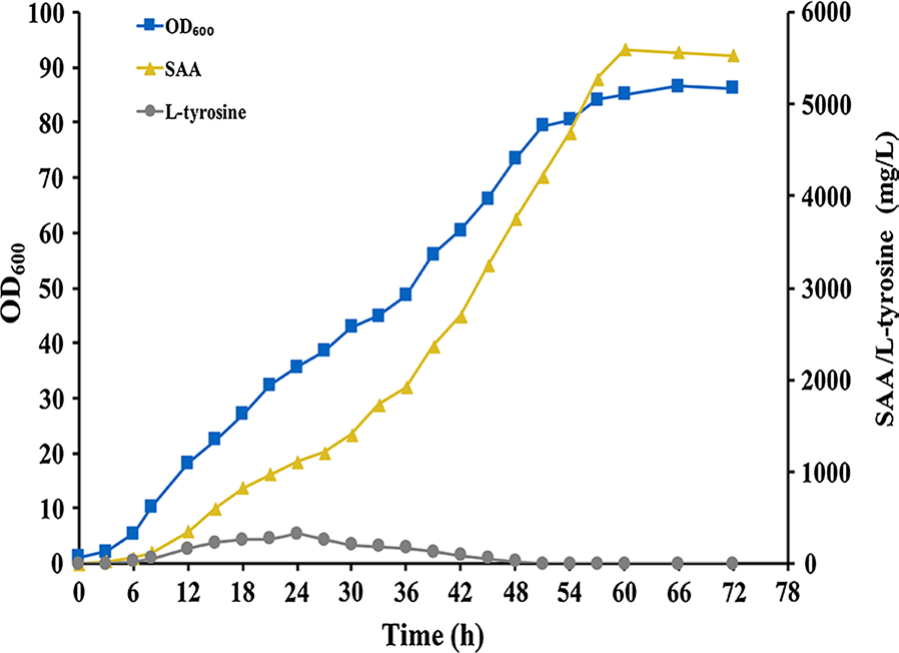
Fed‐batch fermentation of BKD13 with glucose in 5 L bioreactor

To evaluate the performance of plasmid-free and inducer-free SAA producing strain BKD13, the fed-batch fermentation was further carried out in a 5 L bioreactor. Based on concentration of residual glucose, feeding solution was added into the bioreactor to maintain it lower than 1.0 g/L. As shown in Fig. 5, during the fermentation process, the consumption of glucose was used for cell growth as well as for SAA biosynthesis, thus, the production of SAA coupled with the formation of biomass [55]. The final titer of 5.6 g/L SAA with the maximal biomass at OD_600_ ~ 86 was achieved at 60 h. Small amount of precursor l-tyrosine accumulated in the early stage of the fermentation and completely converted into SAA after 51 h, while tiny amount of l-dopa (5.5 mg/L) was detected only before 36 h, and the fermentation broth had never become brown or dark. These results indicated that the plasmid-free and inducer-free strain BKD13 could constitutively produce SAA from glucose. We previously constructed a plasmid-mediated strain BKD5, which produced 7.1 g/L of SAA and accumulated 53.8 mg/L of l-tyrosine in 70 h [12]. Compared to strain BKD5, strain BKD13 seems more efficient for the conversion of l-tyrosine precursor to SAA since no l-tyrosine was detected at the end of fermentation. However, cell growth of strain BKD13 was faster than that of strain BKD5 (OD_600_ ~ 4.0), which might lead to lower titer of SAA. Additionally, one copy of SAA synthetic genes in the chromosome of strain BKD13 might not be sufficient to utilize the central carbon metabolites to synthesize SAA. Amplification of integrated SAA synthetic genes [56] and optimization of fermentation process would further improve SAA production of strain BKD13.

## Conclusions

In this paper, we developed a constitutive SAA-overproducing *E. coli* strain by chromosomal engineering to overcome the drawbacks of plasmid-mediated expression systems. In order to streamline metabolic flux towards precursor l-tyrosine from glucose and eliminate the repression regulation of LacI, module 1 (P_*lacUV5*_-*aroG* ^*fbr*^-*tyrA* ^*fbr*^-*aroE*) replaced *mao*-*paa* cluster of 17.7 kb and module 2 (P_*trc*_-*ppsA*-*tktA*-*glk*) replaced *lacI* gene in strain BAK5 by targeted integration and deletion. Furthermore, we screened the synthetic promoter *5tacs* driving the expression of module 3 (*hpaBC*-*d*-*ldh* ^*Y52A*^) in one operon, which was further inserted at the position between *nupG* and *speC*. The plasmid-free and inducer-free *E. coli* strain BKD13 produced 5.6 g/L SAA from glucose at 60 h in a 5L fed-batch fermentation. Our work showed the opportunities for industrial production of SAA and its derivatives, like rosmarinic acid and salvianolic acids, as a good example without additional antibiotics nor inducers for engineered microbial fermentation.

## Additional file

**Additional file 1: Table S1.** Primers used in this study. **Table S2.** Sequences of three fragments F1, F2 and F3. **Figure S1.** Assembly flowchart of ydbL-M1(P_lacUV5_-aroG^fbr^-tyrA^fbr^-aroE)-Chl-ydbA fragment for replacing *mao*-*paa* cluster of strain BAK5 chromosome. **Figure S2.** Expression plasmid maps of pZL3, pZL4, pZL5, pZL6, pZL9 and pZL10. **Figure S3.** HPLC chromatogram of SAA production by engineered strain.

## Abbreviations

SAA: salvianic acid A
IPTG: isopropyl-β-d-thiogalactopyranoside
PEP: phosphoenolpyruvate
4‐HPP: 4‐hydroxyphenylpyruvic acid
5′-UTR: 5′-untranslated region
*hpaBC*: 4-hydroxyphenylacetate 3-hydroxylase
*d*-*ldh*^*Y52A*^: d-lactate dehydrogenase (Tyr 52 to Ala)

## References

[1] Zhao GR, Xiang ZJ, Ye TX, Juan YJ, Guo ZX (2006). Antioxidant activities of *Salvia miltiorrhiza* and *Panax notoginseng*. Food Chem.

[2] Ling S, Luo R, Dai A, Guo Z, Guo R, Komesaroff PA (2009). A pharmaceutical preparation of *Salvia miltiorrhiza* protects cardiac myocytes from tumor necrosis factor-induced apoptosis and reduces angiotensin II-stimulated collagen synthesis in fibroblasts. Phytomedicine.

[3] Zhao GR, Zhang HM, Ye TX, Xiang ZJ, Yuan YJ, Guo ZX, Zhao LB (2008). Characterization of the radical scavenging and antioxidant activities of danshensu and salvianolic acid B. Food Chem Toxicol.

[4] Huang ZS, Zeng CL, Zhu LJ, Jiang L, Li N, Hu H (2010). Salvianolic acid A inhibits platelet activation and arterial thrombosis via inhibition of phosphoinositide 3-kinase. J Thromb Haemost.

[5] Yang Y, Han Z, Wang Y, Wang L, Pan S, Liang S, Wang S (2015). Plasma metabonomic analysis reveals the effects of salvianic acid on alleviating acute alcoholic liver damage. RSC Adv.

[6] Song Q, Chu X, Zhang X, Bao Y, Zhang Y, Guo H, Liu Y, Liu H, Zhang J, Zhang Y (2016). Mechanisms underlying the cardioprotective effect of salvianic acid A against isoproterenol-induced myocardial ischemia injury in rats: possible involvement of L-type calcium channels and myocardial contractility. J Ethnopharmacol.

[7] Zhou L, Zuo Z, Chow MS (2006). Danshen: an overview of its chemistry, pharmacology, pharmacokinetics, and clinical use. J Clin Pharmacol.

[8] Erkan N, Ayranci G, Ayranci E (2008). Antioxidant activities of rosemary (*Rosmarinus Officinalis* L.) extract, blackseed (*Nigella sativa* L.) essential oil, carnosic acid, rosmarinic acid and sesamol. Food Chem.

[9] Pan LL, Wang J, Jia YL, Zheng HM, Wang Y, Zhu YZ (2015). Asymmetric synthesis and evaluation of danshensu–cysteine conjugates as novel potential anti-apoptotic drug candidates. Int J Mol Sci.

[10] Lam FF, Yeung JH, Chan KM, Or PM (2007). Relaxant effects of danshen aqueous extract and its constituent danshensu on rat coronary artery are mediated by inhibition of calcium channels. Vasc Pharmacol.

[11] Sayyed IA, Sudalai A (2004). Asymmetric synthesis of l-DOPA and (R)-selegiline via, OsO_4_-catalyzed asymmetric dihydroxylation. Tetrahedron: Asymmetry.

[12] Yao YF, Wang CS, Qiao J, Zhao GR (2013). Metabolic engineering of *Escherichia coli* for production of salvianic acid A via an artificial biosynthetic pathway. Metab Eng.

[13] Bai CL, Zhao GR (2015). Separation of salvianic acid A from the fermentation broth of engineered *Escherichia coli* using macroporous resins. J Sep Sci.

[14] Friehs K (2004). Plasmid copy number and plasmid stability. Adv Biochem Eng Biot.

[15] Blazeck J, Alper HS (2013). Promoter engineering: recent advances in controlling transcription at the most fundamental level. Biotechnol J.

[16] Matsuda F, Ishii J, Kondo T, Ida K, Tezuka H, Kondo A (2013). Increased isobutanol production in *Saccharomyces cerevisiae* by eliminating competing pathways and resolving cofactor imbalance. Microb Cell Fact.

[17] Jong BWD, Shi S, Siewers V, Nielsen J (2014). Improved production of fatty acid ethyl esters in *Saccharomyces cerevisiae* through up-regulation of the ethanol degradation pathway and expression of the heterologous phosphoketolase pathway. Microb Cell Fact.

[18] Borodina I, Kildegaard KR, Jensen NB, Blicher TH, Maury J, Sherstyk S, Schneider K, Lamosa P, Herrgård MJ, Rosenstand I (2015). Establishing a synthetic pathway for high-level production of 3-hydroxypropionic acid in *Saccharomyces cerevisiae* via β-alanine. Metab Eng.

[19] Sun J, Shao Z, Zhao H, Nair N, Wen F, Xu JH, Zhao H (2012). Cloning and characterization of a panel of constitutive promoters for applications in pathway engineering in *Saccharomyces cerevisiae*. Biotechnol Bioeng.

[20] Dai Z, Liu Y, Zhang X, Shi M, Wang B, Wang D, Huang L, Zhang X (2013). Metabolic engineering of *Saccharomyces cerevisiae* for production of ginsenosides. Metab Eng.

[21] Galanie S, Smolke CD (2015). Optimization of yeast-based production of medicinal protoberberine alkaloids. Microb Cell Fact.

[22] Brown S, Clastre M, Courdavault V, O’Connor SE (2015). De novo production of the plant-derived alkaloid strictosidine in yeast. Proc Natl Acad Sci USA.

[23] Lim CG, Fowler ZL, Hueller T, Schaffer S, Koffas MAG (2011). High-yield resveratrol production in engineered *Escherichia coli*. Appl Environ Microbiol.

[24] Alper H, Fischer C, Nevoigt E, Stephanopoulos G (2005). Tuning genetic control through promoter engineering. Proc Natl Acad Sci USA.

[25] Tong YJ, Ji XJ, Shen MQ, Liu LG, Nie ZK, Huang H (2016). Constructing a synthetic constitutive metabolic pathway in *Escherichia coli* for (R, R)-2,3-butanediol production. Appl Microbiol Biotechnol.

[26] Menezes AC, Suzuki MF, Oliveira JE, Ribela MT, Furigo IC, Donato J, Bartolini P, Soares CR (2016). Expression, purification and characterization of the authentic form of human growth hormone receptor antagonist G120R-hGH obtained in *Escherichia coli* periplasmic space. Protein Expr Purif.

[27] Paddon CJ, Westfall PJ, Pitera DJ, Benjamin K, Fisher K, Mcphee D, Leavell MD, Tai A, Main A, Eng D (2013). High-level semi-synthetic production of the potent antimalarial artemisinin. Nature.

[28] Chen Y, Xiao W, Wang Y, Liu H, Li X, Yuan Y (2016). Lycopene overproduction in *Saccharomyces cerevisiae* through combining pathway engineering with host engineering. Microb Cell Fact.

[29] Lemuth K, Steuer K, Albermann C (2011). Engineering of a plasmid-free *Escherichia coli* strain for improved in vivo biosynthesis of astaxanthin. Microb Cell Fact.

[30] Cui YY, Chen L, Zhang YY, Jian H, Liu JZ (2014). Production of shikimic acid from *Escherichia coli* through chemically inducible chromosomal evolution and cofactor metabolic engineering. Microb Cell Fact.

[31] Li Y, Lin Z, Huang C, Zhang Y, Wang Z, Tang YJ, Chen T, Zhao X (2015). Metabolic engineering of *Escherichia coli* using CRISPR-Cas9 meditated genome editing. Metab Eng.

[32] Datsenko KA, Wanner BL (2000). One-step inactivation of chromosomal genes in *Escherichia coli* K-12 using PCR products. Proc Natl Acad Sci USA.

[33] Neidhardt FC, Bloch PL, Smith DF (1974). Culture medium for enterobacteria. J Bacteriol.

[34] Gosset G (2009). Production of aromatic compounds in bacteria. Curr Opin Biotechnol.

[35] Patnaik R, Zolandz RR, Green DA, Kraynie DF (2008). l-Tyrosine production by recombinant *Escherichia coli*: fermentation optimization and recovery. Biotechnol Bioeng.

[36] Rodriguez A, Martínez JA, Flores N, Escalante A, Gosset G, Bolivar F (2014). Engineering *Escherichia coli* to overproduce aromatic amino acids and derived compounds. Microb Cell Fact.

[37] Lütkeeversloh T, Stephanopoulos G (2008). Combinatorial pathway analysis for improved l-tyrosine production in *Escherichia coli*: identification of enzymatic bottlenecks by systematic gene overexpression. Metab Eng.

[38] Santos CN, Xiao W, Stephanopoulos G (2012). Rational, combinatorial, and genomic approaches for engineering l-tyrosine production in *Escherichia coli*. Proc Natl Acad Sci USA.

[39] Juminaga D, Baidoo EE, Reddingjohanson AM, Batth TS, Burd H, Mukhopadhyay A, Petzold CJ, Keasling JD (2012). Modular engineering of l-tyrosine production in *Escherichia coli*. Appl Environ Microbiol.

[40] Kang SY, Choi O, Lee JK, Ahn JO, Ahn JS, Hwang BY, Hong YS (2015). Artificial de novo biosynthesis of hydroxystyrene derivatives in a tyrosine overproducing *Escherichia coli* strain. Microb Cell Fact.

[41] Díaz E, Ferrández A, Prieto MA, García JL (2001). Biodegradation of aromatic compounds by *Escherichia coli*. Microbiol Mol Biol Rev.

[42] Dvorak P, Chrast L, Nikel PI, Fedr R, Soucek K, Sedlackova M, Chaloupkova R, Lorenzo VD, Prokop Z, Damborsky J (2015). Exacerbation of substrate toxicity by IPTG in *Escherichia coli* BL21(DE3) carrying a synthetic metabolic pathway. Microb Cell Fact.

[43] Kim SC, Min BE, Hwang HG, Sang WS, Jung GY (2014). Pathway optimization by re-design of untranslated regions for l-tyrosine production in *Escherichia coli*. Sci Rep.

[44] Lim JH, Seo SW, Kim SY, Jung GY (2013). Model-driven rebalancing of the intracellular redox state for optimization of a heterologous n-butanol pathway in *Escherichia coli*. Metab Eng.

[45] Kwak DH, Lim HG, Yang J, Seo SW, Jung GY (2017). Synthetic redesign of *Escherichia coli* for cadaverine production from galactose. Biotechnol Biofuels.

[46] Boer HAD, Comstock LJ, Vasser M (1983). The tac promoter: a functional hybrid derived from the trp and lac promoters. Proc Natl Acad Sci USA.

[47] Lu W, Shi Y, He S, Fei Y, Yu K, Yu H (2013). Enhanced production of CoQ 10 by constitutive overexpression of 3-demethyl ubiquinone-9 3-methyltransferase under tac promoter in *Rhodobacter sphaeroides*. Biochem Eng J.

[48] Aghaabdollahian S, Rabbani M, Ghaedi K (2014). Sadeghi HMM. Molecular cloning of Reteplase and its expression in *E. coli* using tac promoter. Adv Biomed Res.

[49] Seo SW, Yang JS, Kim I, Yang J, Min BE, Kim S, Jung GY (2013). Predictive design of mRNA translation initiation region to control prokaryotic translation efficiency. Metab Eng.

[50] Muñoz AJ, Hernández-Chávez G, Anda RD, Martínez A, Bolívar F, Gosset G (2011). Metabolic engineering of l-3,4-dihydroxyphenylalanine (l-DOPA) synthesis from glucose. J Ind Microbiol Biotechnol.

[51] Maeda M, Shimada T, Ishihama A (2015). Strength and regulation of seven rRNA promoters in *Escherichia coli*. PLoS ONE.

[52] Wu H, Wang H, Chen J, Chen GQ (2014). Effects of cascaded *vgb* promoters on poly(hydroxybutyrate) (PHB) synthesis by recombinant *Escherichia coli* grown micro-aerobically. Appl Microbiol Biotechnol.

[53] Li M, Wang J, Geng Y, Li Y, Qian W, Liang Q, Qi Q (2012). A strategy of gene overexpression based on tandem repetitive promoters in *Escherichia coli*. Microb Cell Fact.

[54] Bryant JA, Sellars LE, Busby SJ, Lee DJ (2014). Chromosome position effects on gene expression in *Escherichia coli* K-12. Nucleic Acids Res.

[55] De Hollander JA (1993). Kinetics of microbial product formation and its consequences for the optimization of fermentation processes. Antonie Van Leeuwenhoek.

[56] Tyo KEJ, Ajikumar PK, Stephanopoulos G (2009). Stabilized gene duplication enables long-term selection-free heterologous pathway expression. Nat Biotechnol.

