# Supplementary material for: Chromosome engineering of Escherichia coli for constitutive production of salvianic acid A
Source: Microb Cell Fact. 2017 May 16;16:84. doi: 10.1186/s12934-017-0700-2 (PMC5434548; doi:10.1186/s12934-017-0700-2)
Supplement: Supplementary file 1 — Additional file 1: Table S1. Primers used in this study. Table S2. Sequences of three fragments F1, F2 and F3. Figure S1. Assembly flowchart of ydbL-M1(PlacUV5-aroGfbr-tyrAfbr-aroE)-Chl-ydbA fragment for replacing mao-paa cluster of strain BAK5 chromosome. Figure S2. Expression plasmid maps of pZL3, pZL4, pZL5, pZL6, pZL9 and pZL10. Figure S3. HPLC chromatogram of SAA production by engineered strain. [file 12934_2017_700_MOESM1_ESM.docx]

**Additional file 1**

**Chromosome engineering of *Escherichia coli* for constitutive production of salvianic acid A**

Liang Zhou ^1,2,3^, Qi Ding ^1,2,4^, Guo-Zhen Jiang^1,2,3^, Zhen-Ning Liu^1,2,3^, Hai-Yan Wang^1,2,5^, Guang-Rong Zhao^1,2,3^ *

^1^Department of Pharmaceutical Engineering, School of Chemical Engineering and Technology, Tianjin University, Tianjin, 300072, China

^2^Key Laboratory of Systems Bioengineering (Ministry of Education), Tianjin University

^3^SynBio Research Platform, Collaborative Innovation Center of Chemical Science and Engineering (Tianjin)

^4^ Present address: College of Chemical and Biological Engineering, Zhejiang University, No.38 Zhe da Road. Hangzhou 310027, China

^5^ Present address: Yangtze River Pharmaceutical Group Co, Ltd. 1 Yangtze River South Road, Taizhou 225321, China

Email addresses:

[lzhou@tju.edu.cn](mailto:lzhou@tju.edu.cn)

[dq_339799811@qq.com](mailto:dq_339799811@qq.com)

[guozhenj@tju.edu.cn](mailto:guozhenj@tju.edu.cn)

[1040234410@qq.com](mailto:1040234410@qq.com)

[827219958@qq.com](mailto:827219958@qq.com)

*Correspondence: [grzhao@tju.edu.cn](mailto:grzhao@tju.edu.cn); Tel: +86-22-85356580; Fax: +86-22-27403389

**Table S1.**  Primers used in this study.

| **Name** | **Sequence (5’-3’)** |
| --- | --- |
| rrnB F | CCGGGGCCCGCCAGGAGCTGAACAATTA |
| rrnB R | CCCAAGCTTTGGTGGCGCATTATAGGGA |
| hpaBC F | CCCAAGCTTACATCCCGTCAAAGGAGCATCGACCATGAAACCAGAAGATTTCCGCG |
| hpaBC R | GGACTAGTTTAAATCGCAGCTTCCATTTCC |
| d-ldh^Y52A^ F | CCGGAATTCCCACAATACCAAAGGAGCATCTAACATGAAAATTATTGCCTATGCTGTAC |
| d-ldh^Y52A^ R | CGCGGATCCCAGTGATAATCGAGATTAGTCAAACT |
| ydbL F | GAAGTCGCTGCACCTAAGGAACCGGAGAGGCGGTTTGCGTATTGGG |
| ydbL R | CGCCTTCTCCTGTAGCGTTATCCCGATAT |
| M1 F | CCCAATACGCAAACCGCCTCTCC |
| M1 R | TCCCAATACGCAAACCGCCTCTCC |
| Chl F | GGAGAGGCGGTTTGCGTATTGGGAGTGTAGGCTG GAGCTGCTTC |
| Chl R | GTCTCGAATTTGTATTGAGACGAAATATTA ATGGGAATTAGCCATGGTCC |
| ydbA F | TAATATTTCGTCTCAATACAAATTC |
| ydbA R | AGGCCGTGATTAACGCAGCAGCG |
| paaZ F | ATGCAGCAGTTAGCCAGTTTC |
| paaZ R | TTAATCGACAAAATCACCGTGC |
| lacZ F | CGGCATTGGCTGGGTCGCGG |
| lacZ R | CGTTAATTACACTCAATCGAGTGCAGGTGAAACTGACCGATAAGCCG |
| M2 F | CGGCTTATCGGTCAGTTTCACCTG |
| M2 R | TGCAGCGACTGCACGGTGCACC |
| Chl F1 | GGTGCACCGTGCAGTCGCTGCAGTGTAGGCTG GAGCTGCTTC |
| Chl R1 | GTACGTAAATGCAACTGTGGTAGCTTACCGCGATGGGAATTAGCCATGGTC |
| mhpR F | CGCGGTAAGCTACCACAGTTGCATTTAC |
| mhpR R | CGTTACCACCATGCGCTTTGGTGAGTGGC |
| nupG F | GTTCATTCTGACCATCCCG |
| nupG R | CAAATTCAGCCGATAGCGGAACGGGAAGGCACCCGTTTTTCTTTGCGTA |
| M3 F | GCCTTCCCGTTCCGCTATC |
| M3 R | CTGCGCTAGTAGACGAGTCC |
| Chl F2 | GCCAGCACATGGACTCGTCTACTAGCGCAGGTGTAGGCTGGAGCTGCTTC |
| Chl R2 | TAAAAAAAACGGGTCACCTTCTGGCG ATGGGAATTAGCCATGGTCC |
| SpeC F | CGCCAGAAGGTGACCCGTT |
| SpeC R | TTGAGGATGACTCGCCGCT |

The 5’UTR sequences of *hpaBC* and *d-ldh^Y52A^* were underlined in corresponding primer.

**Table S2.** Sequences of three fragments F1, F2 and F3.

| **Name** | **Sequences** |
| --- | --- |
| Fragment 1 (F1) containing two  *BBaJ23100* promoters | CCGGGGCCCTTGACGGCTAGCTCAGTCCTAGGTACAGTGCTAGCCCCAAGCTTGGGCATGCCATGGCATGCGGACTAGTCCGAAATGTTAACGGCCGCATAATCGAAATTTGACGGCTAGCTCAGTCCTAGGTACAGTGCTAGCCCGGAATTCCGGCATGCCATGGCATGCGCGGATCCGCGCCAGGCATCAAATAAAACGAAAGGCTCAGTCGAAAGACTGGGCCTTTCGTTTTATCTGTTGTTTGTCGGTGAACGCTCTCTACTAGAGTCACACTGGCTCACCTTCGGGTGGGCCTTTCTGCGTTTATACTCGAGCGG |
| Fragment 2 (F2)  containing two  *tac* promoters | CCGGGGCCCTTGACAATTAATCATCGGCTCGTATAATGTGTGGAATTGTGCCCAAGCTTGGGCATGCCATGGCATGCGGACTAGTCCGAAATGTTAACGGCCGCATAATCGAAATTTGACAATTAATCATCGGCTCGTATAATGTGTGGAATTGTGCCGGAATTCCGGCATGCCATGGCATGCGCGGATCCGCGCCAGGCATCAAATAAAACGAAAGGCTCAGTCGAAAGACTGGGCCTTTCGTTTTATCTGTTGTTTGTCGGTGAACGCTCTCTACTAGAGTCACACTGGCTCACCTTCGGGTGGGCCTTTCTGCGTTTATACTCGAGCGG |
| Fragment 3 (F3)  containing  5*tacs* promoter | CCGGGGCCC【TTGACAATTAATCATCGGCTCGTATAATGTGTGGAATT  GTG】×5 CCCAAGCTTGGGCATGCCATGGCATGCCGACTAGTCGGA AATGTTAACGGCCGCATAATCGAAATCGCGAATTCGCGCATGCCATGGCATGCCGGGATCCGCGCCAGGCATCAAATAAAACGAAAGGCTCAGTCGAAAGACTGGGCCTTTCGTTTTATCTGTTGTTTGTCGGTGAACGCTCTCTACTAGAGTCACACTGGCTCACCTTCGGGTGGGCCTTTCTGCGTTTATA CTCGAGCGG |

The background of promoter sequence was highlighted in gray color.

**Figure S1.** Assembly flowchart of ydbL-M1(P_lacUV5_-aroG^fbr^-tyrA^fbr^-aroE)-Chl-ydbA fragment for replacing *mao-paa* cluster of strain BAK5 chromosome.

**Figure S2.** Expression plasmid maps of pZL3, pZL4, pZL5, pZL6, pZL9 and pZL10.


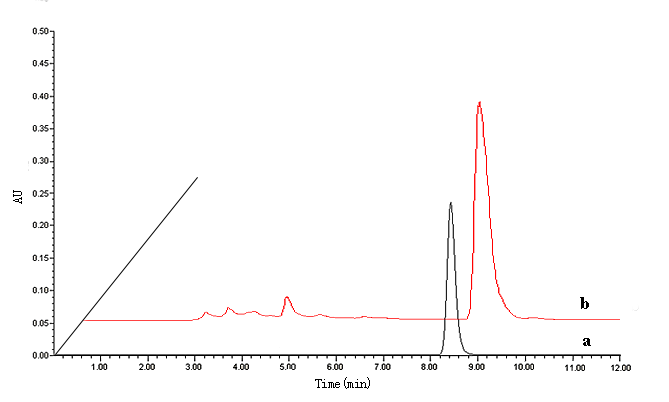


Figure S3: HPLC chromatogram of SAA production by engineered strain.

(a): Standard SAA; (b): Fermentation supernatant.
